# Strengthening Mechanism of Al/Ni Multilayers with Negative Enthalpy of Mixing

**DOI:** 10.1021/acs.nanolett.5c02939

**Published:** 2025-08-19

**Authors:** Xi Li, Nicolas J. Peter, Marilaine Moreira de Lima, Sebastian Matthes, Peter Schaaf, Ruth Schwaiger

**Affiliations:** † 249097 Institute of Energy Materials and Devices, Structure and Function of Materials (IMD-1), Forschungszentrum Jülich GmbH, 52425 Jülich, Germany; ‡ 26559 Chair Materials for Electrical Engineering and Electronics, Institute of Materials Science and Engineering, Institute of Micro and Nanotechnologies MacroNano, TU Ilmenau, Gustav-Kirchhoff-Str. 5, 98693 Ilmenau, Germany

**Keywords:** nanoscale multilayer, interface strengthening, enthalpy of mixing, intermetallic

## Abstract

The interface strengthening effect in nanoscale metallic multilayers is influenced by the enthalpy of mixing, which governs the chemical distribution and interface microstructure. In this study, Al/Ni multilayers were fabricated by magnetron sputter deposition, exhibiting an ultrahigh peak hardness of 9.5 GPa—the highest reported for face-centered cubic multilayer systems. Advanced electron microscopy revealed extensive interdiffusion at the Al/Ni interfaces and the formation of intermetallic bonds at both interfaces and grain boundaries. A modified confined layer slip model is proposed, accounting for energy changes associated with trailing dislocations propagating through interfaces or grain boundaries due to intermetallic bond formation. The model aligns closely with experimental data, demonstrating that intermetallic bond formation in Al/Ni multilayers significantly enhances interface strengthening, counteracting the weakening effects of interface diffusion. This mechanism may also account for the high peak hardness observed in other multilayer systems with large negative enthalpies of mixing.

## Graphical abstract

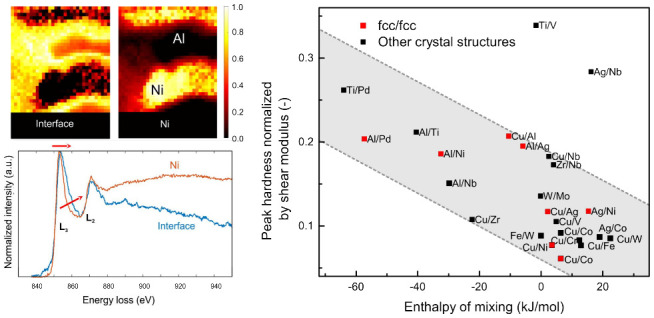

Nanoscale metallic multilayers have attracted attention for their ultrahigh strengths, which approach theoretical limits. Researchers have developed systems of face-centered cubic (fcc), body-centered cubic (bcc), and hexagonal close-packed (hcp) materials to explore how interface types influence mechanical behavior.^1–3^ Common combinations include fcc/fcc,^4–7^ fcc/bcc,^4,8–10^ hcp/bcc,^11,12^ and fcc/hcp^13,14^ systems. Materials are selected based on factors such as modulus mismatch,^15^ lattice parameter mismatch,^16,17^ and stacking fault energy^18^ to systematically explore interface strengthening. A major focus is on size-dependent strengthening mechanisms that vary with the layer thickness (*h*). For *h* > 50 nm, the Hall–Petch relation (*H* = *H*_0_ + *kh* ^–1/2^) applies, where *H*_0_ describes film hardness due to lattice friction and *k* is the Hall–Petch slope.^19^ Here, deformation is dominated by dislocation pile-ups at interfaces.^1,20^ When 10 nm < *h* ≤ 50 nm, pile-ups are suppressed, and single dislocation mechanisms prevail.^21^ Peak strength typically occurs at *h* ≈ 5 nm; below this, softening can occur due to dislocation transmission across interfaces, especially in coherent systems.^1^

Atomically sharp interfaces with a large lattice mismatch impede dislocation motion. Interface stability is typically ensured using material pairs with positive or near-zero enthalpies of mixing, such as Cu/Ni,^4^ Cu/Ag,^6^ and Cu/Nb.^4^ Also, systems with negative enthalpy of mixing, e.g., Cu/Al,^7,22^ Al/Pd,^23^ Ti/Nb,^24^ and Pd/Ti,^25^ have been explored, although not all address how enthalpy influences strengthening. Negative enthalpy can promote interdiffusion and intermetallic formation, which may enhance stiffness and strength but also increase brittleness.^26^

Experiments revealed mixed effects. In Cu/Al multilayers, interdiffusion at interfaces stabilized stacking faults and increased hardness,^22^ while annealing promoted intermetallic growth and further strengthening. By contrast, Cu/Nb systems with graded interfaces exhibited superior yield and flow strengths versus sharp ones.^27^ Ti/Nb interfaces exhibiting intermixing demonstrated a noticeable enhancement in hardness, likely due to the complex structure of the metastable interfaces.^24^ These results highlight the complex role of the enthalpy of mixing in interfacial strengthening, particularly for highly reactive systems.

This study investigates Al/Ni multilayers to clarify how interfaces with negative enthalpies of mixing affect strengthening. Detailed microstructural and chemical analyses were conducted to explain the high peak strength observed. Interface barrier and shear stresses were assessed by using the confined layer slip model. Comparisons with other systems reveal how the chemical activity influences mechanical performance in nanoscale multilayers.

Al/Ni multilayer thin films, 1 μm thick in total, were deposited on Si wafers with 250 nm SiO_2_ passivation by using DC magnetron sputtering. The individual Al and Ni layer thicknesses *h* were set to 5, 10, 25, 50, 100, and 250 nm, yielding six distinct samples. Microstructures were characterized by using multiple techniques. X-ray diffraction (XRD) on an Empyrean X-ray diffractometer with a copper source identified phases, crystallography, and texture. Transmission electron microscopy (TEM) was used to assess the microstructure; layer thickness and composition were characterized with aberration-corrected Titan Themis 80–300 scanning TEM (STEM, Thermo Fischer Scientific, USA) at 300 kV. High-angle annular dark-field (HAADF) imaging was performed with a semiconvergence angle of 23.7 mrad and semicollection angle of 70–200 mrad. Energy-dispersive X-ray spectroscopy (EDS), using a four-quadrant SuperX detector, determined the composition. For *h* = 25 nm, electron energy loss spectroscopy (EELS) was used to study the interface, with spectrum maps collected over a 65 × 65 nm^2^ field-of-view at 2 nm pixel resolution. Non-negative matrix factorization (NMF)^28^ decomposed these maps into dominant components with corresponding spectra and spatial distributions. Grain sizes were determined via dark-field TEM and selected area electron diffraction (SAED), while texture was analyzed by radially integrating SAED diffractograms and comparing with XRD. High-resolution TEM (HRTEM) at 300 kV resolved atomic-scale interface structures and phases. Cross-sectional TEM lamellae were prepared by a Xe-plasma focused ion beam (PFIB, Thermo Fischer Scientific, USA), with a final 5 kV polish to minimize ion damage.

Nanoindentation was performed on as-deposited films using a Nanoindenter XP instrument (MTS Systems Corp., Eden Prairie, MN, USA) with a diamond Berkovich tip in continuous stiffness measurement (CSM) mode. At least 10 indents per sample were performed to a maximum indentation depth of 1 μm at a constant strain rate *Ṗ*/*P* of 0.05 s^–1^. Hardness and modulus were evaluated via the Oliver-and-Pharr method,^29,30^ with data from the 100–200 nm depth range to minimize substrate effects.^31^ All tests were conducted at room temperature to prevent additional interdiffusion and phase transformation from thermal effects.^32^

X-ray diffractograms of Al/Ni multilayers show a mildly textured Ni(1 1 1)/Al(2 0 0) peak along with Ni(2 2 0), with no evidence of Al–Ni intermetallic phases. The lattice misfit between Al and Ni, calculated from XRD peak positions, is 13.0%. Full XRD results are provided in the Supporting Information. Layer thicknesses and grain sizes, used in subsequent calculations, were measured from cross-sectional TEM images, also detailed in the Supporting Information. Additionally, SAED patterns obtained from the same cross-sectional TEM analysis did not indicate any intermetallic compounds. HAADF-STEM micrographs and corresponding STEM-EDS analyses confirm alternating Ni and Al layers in *h* = 5 and 50 nm samples (Figures 1a–f). EDS line profiles reveal gradual compositional transitions at interfaces, attributed to intermixing or roughness. For *h* = 5 nm, strong intermixing yields Ni-rich Al (50:50 at. %) and Al-rich Ni (85:15 at. %) layers. With increasing *h*, the peak concentration rises, becoming pure Al or Ni at *h* ≥ 50 nm. Average peak concentrations of Al and Ni, taken from the maxima in EDS profiles, define the Al/Ni peak concentration ratio (Figure 1g), which indicates deviations from the ideal composition (ratio = 1). Ratios decrease for *h* ≤ 25 nm, confirming significant intermixing, while samples with *h* ≥ 50 nm retain regions of compositional purity beyond the intermixed interface. In the *h* = 5 nm sample, the Al peak concentration reaches only 50% (Figure 1e), explaining the weak or absent Al diffraction peaks in the XRD.

**Figure 1:**
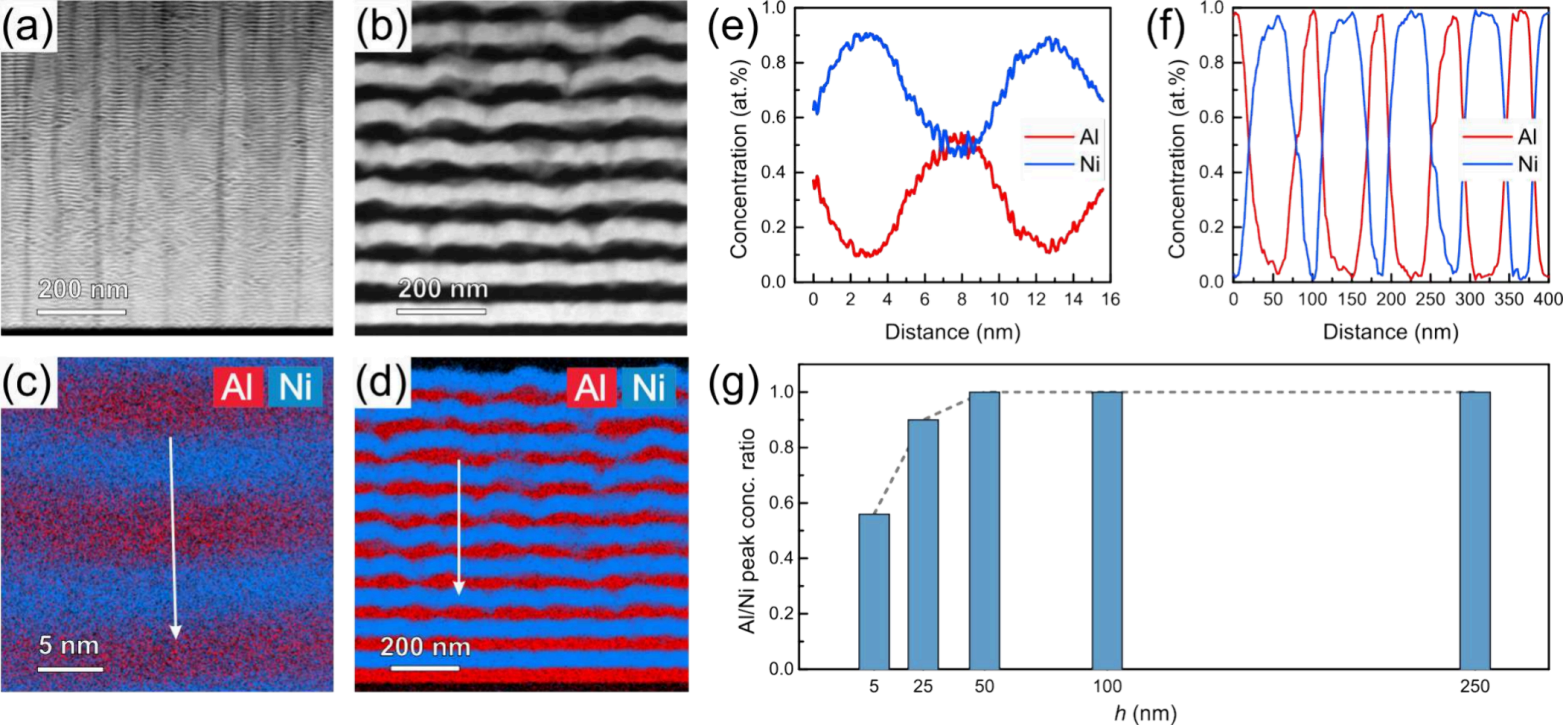
Cross-sectional HAADF-STEM micrographs of Al/Ni multilayers with nominal layer thicknesses of (a) 5 nm and (b) 50 nm. STEM-EDS elemental maps for (c) 5 nm and (d) 50 nm samples. (e, f) Corresponding line profiles taken along the arrows in (c) and (d), respectively. (g) Al/Ni peak concentration ratio plotted as a function of nominal layer thickness.

The bonding state of Ni at the Al/Ni interfaces was examined via EELS for the *h* = 25 nm sample (Figure 2a–c), focusing on both well-defined layers and intermediate-contrast regions suggesting intermixing (Figure 2a). Component analysis (Figure 2b) of the EELS spectra (Figure 2c) revealed two components: one corresponding to pure Ni and the other to interfacial regions. Normalized Ni-*L*_3,2_ edge spectra (Figure 2c) show an onset at ∼850 eV; the interfacial component exhibits a broadened and slightly shifted *L*_3_ white line, resembling Ni_3_Al and NiAl intermetallics,^33,34^ though not definitive of intermetallic formation. Instead, bonding appears intermetallic-like and is present at most interfaces in the *h* = 25 nm sample. The interface component reduces the width of the Al layer more significantly than does the Ni layer, suggesting Ni diffusion into Al. For *h* = 250 nm, interfaces are sharper, occasionally showing B2-NiAl intermetallic pockets. As shown in Figure 2d, one such B2-NiAl intermetallic pocket was observed along micrometer-scale segments of the interface. In summary, samples with smaller *h* exhibit 4 nm wide intermixed regions with intermetallic-like bonding, while those with larger *h* retain interfaces with only sporadic intermetallic features.

**Figure 2:**
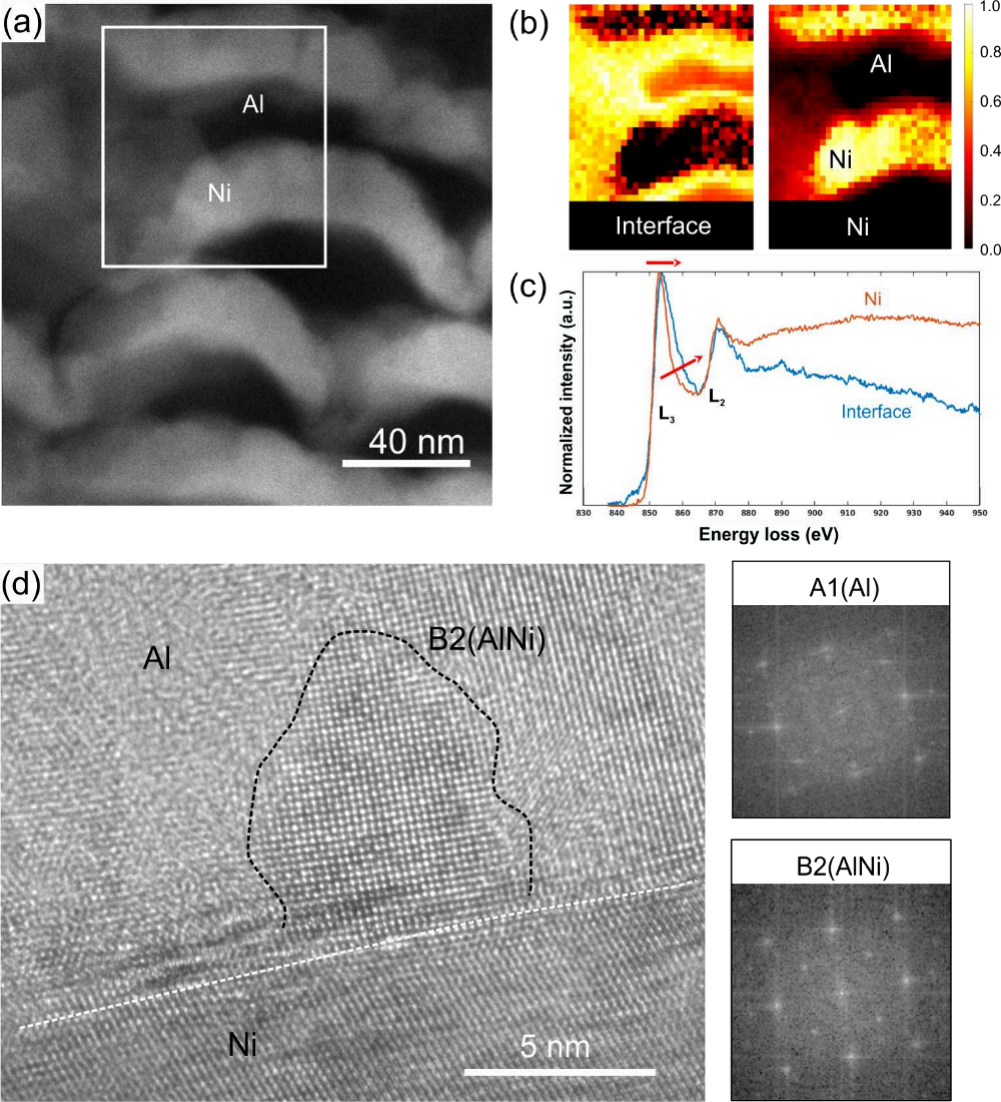
(a) HAADF-STEM micrograph of the sample with *h* = 25 nm. (b) EELS component maps showing interfacial and pure Ni regions. (c) Ni-*L*_3,2_ and interface-*L*_3,2_ spectra for EELS components corresponding to the mapped regions. (d) HRTEM image of the Al/Ni interface in the *h* = 250 nm sample, highlighting a B2-ordered intermetallic region. The white dashed line marks the Al–Ni interface; the black dashed line indicates the boundary of the B2-ordered intermetallic region. Fast Fourier transform (FFT) patterns of the Al and B2 regions are shown on the right.

The mechanical properties of the Al/Ni multilayers were characterized via nanoindentation. Figure 3 shows the elastic modulus and hardness as functions of λ^–1/2^, where λ represents either layer thickness *h* (full black squares) or grain size (open black squares). The elastic modulus displays a bimodal trend: for λ ≥ 50 nm, values range from 136.7 to 139.5 GPa, approaching the rule of mixtures (ROM) upper bound (134.9 GPa). For λ ≤ 50 nm, the modulus increases with decreasing λ, peaking at 157.63 GPa. This enhancement is attributed to:

(i) Composition ratio: EDS mapping reveals a near 1:1 Al-to-Ni ratio for 50 nm ≤ λ ≤ 250 nm, decreasing for λ ≤ 50 nm. A higher Ni content at smaller λ increases the modulus.
(ii) Texture effect: XRD and TEM show (1 1 1) and (2 0 0) texture at λ = 5 nm, corresponding to higher biaxial moduli than in isotropic polycrystals.
(iii) Intermetallic bonding: EELS of the λ = 25 nm sample suggests Al–Ni bonding with intermetallic-like characteristics, which typically yield a higher modulus than solid solutions.^35^ Interfaces are preferential sites for interdiffusion and phase transformations.^36^ EDS profiles support interdiffusion, although XRD did not detect intermetallic phases, likely due to limited volume and crystallite size.

**Figure 3:**
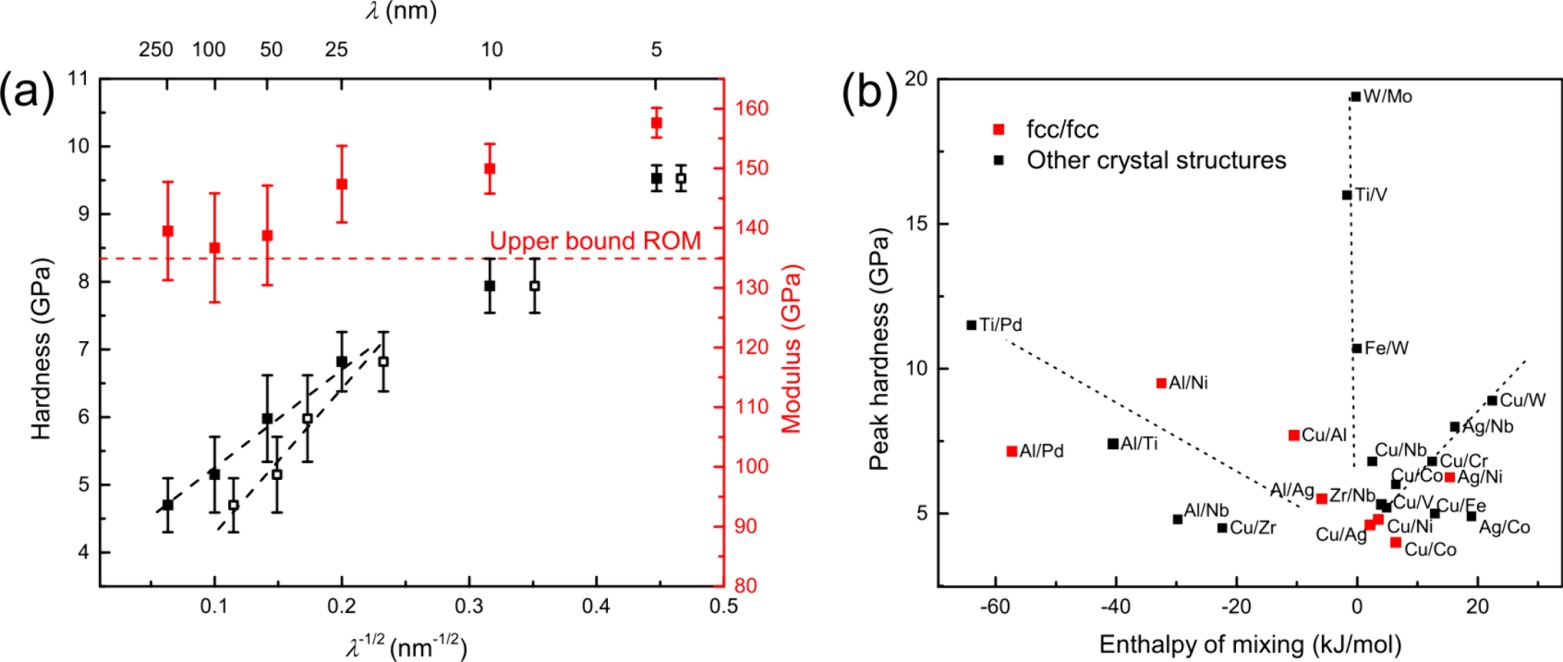
(a) Modulus and hardness of Al/Ni multilayers plotted against λ^–1/2^, where λ represents the layer thickness (full symbols) and grain size (open symbols). In the range 50 ≤ λ ≤ 250 nm, hardness follows a linear Hall–Petch trend. (b) Relationship between peak hardness and the enthalpy of mixing in various metallic multilayers. Data sources include: Ag/Al,^18^ Cu/V,^37^ Cu/Nb,^37^ Cu/Cr,^37^ Cu/W,^37^ Ag/Nb,^37^ Cu/Fe,^10^ Ag/Ni,^38^ Al/Pd,^23^ Cu/Al,^22^ Cu/Co,^39^ Cu/Ni,^1^ Cu/Ag,^1^ Ag/Co,^9^ W/Mo,^9^ Ti/Pd,^25^ Ti/V,^25^ Fe/W,^40^ Cu/Zr,^41^ Zr/Nb,^11^ and Al/Ag.^18^

The hardness of the multilayers exhibits a strong size dependence. For full symbols (λ = *h*), hardness increases linearly with λ^–1/2^ in the range 50–250 nm, following the Hall–Petch relation, with a fit yielding *H*_0_ = 3.62 GPa and $k=14.7\;\mathrm{GPa}\cdot \sqrt{\mathrm{nm}}$ . Below 50 nm, the hardness continues to rise but deviates from linearity, peaking at 9.5 GPa for λ = 5 nm. When *h* ≥ 50 nm, the grain size in the Al layer (i.e., width of grains) is smaller than *h* (see Supporting Information) and governs the size effect.^42^ A Hall–Petch fit in this regime gives *H*_0_ = 2.16 GPa and $k=21.4\;\mathrm{GPa}\cdot \sqrt{\mathrm{nm}}$ .

The 9.5 GPa peak hardness is the highest reported among fcc/fcc multilayers. While fcc/fcc interfaces typically offer dislocation barriers weaker than those of fcc/bcc or fcc/hcp systems due to slip alignment, higher strength can occur when the enthalpy of mixing is negative. Figure 3b compiles multilayer data, showing the link between peak hardness and enthalpy of mixing, with fcc/fcc systems in red. Enthalpy values are calculated via Miedema’s model.^43,44^ In multilayers, negative enthalpy promotes interdiffusion, potentially weakening strengthening from the modulus mismatch (Chu-Barnett model^15^) and lattice mismatch by widening misfit dislocation spacing.^45^ Nevertheless, Figure 3b shows that systems with a more negative enthalpy often achieve a higher peak hardness, while those with a positive or near-zero enthalpy show no clear trend. Elastic modulus mismatch also contributes to interface barrier strength and is strongly influenced by interfacial composition in Al/Ni multilayers. EDS results confirm interdiffusion at Al/Ni interfaces. To evaluate modulus-mismatch strengthening at these diffuse interfaces, we follow the Chu-Barnett model.^15^ Let *C*1 and *C*2 be the Al concentrations in adjacent layers. Assuming a constant composition gradient *Ċ* (independent of *h* under fixed deposition conditions^46^), interface width *w* is defined as (*C*1 – *C*2)/*Ċ*. For *h* ≥ 50 nm, EDS approximates *C*1 = 1 and *C*2 = 0, indicating pure Al and Ni, with interface width *w*_0_. For smaller *h*, even below *w*_0_, *C*1 and *C*2 deviate from 1 and 0, representing fully interdiffused layers without distinct Al or Ni. EDS confirms this trend, with peak compositions decreasing for *h* ≤ 25 nm, although actual gradients may be underestimated due to resolution limits. When dislocations cross interfaces of finite width, the maximum shear stress τ_max_ occurs at the interface center and is given by

1 $$\tau_{\max}=\tau_{0}+\tau_{core}+\tau_{st}$$

where τ_0_ is the average shear stress for slip in a homogeneous material, and τ_core_ and τ_st_ arise from dislocation core and strain field effects, respectively.

Figure 4a shows the critical shear stress enhancement τ_max_^*^ – τ_0_^*^ at the yield for interface widths from 1 to 50 nm. As *w*_0_ increases and *h* decreases, τ_max_^*^ – τ_0_^*^ declines. Assuming that *w*_0_ is constant between 25 and 50 nm, the strengthening contribution is 0.05 GPa for *h* ≥ 50 nm and decreases for smaller *h*. This corresponds to τ* values below 0.001 μ, about one-tenth of the Koehler stress (0.01 μ), which estimates strengthening from modulus mismatch at sharp interfaces.^47^

**Figure 4:**
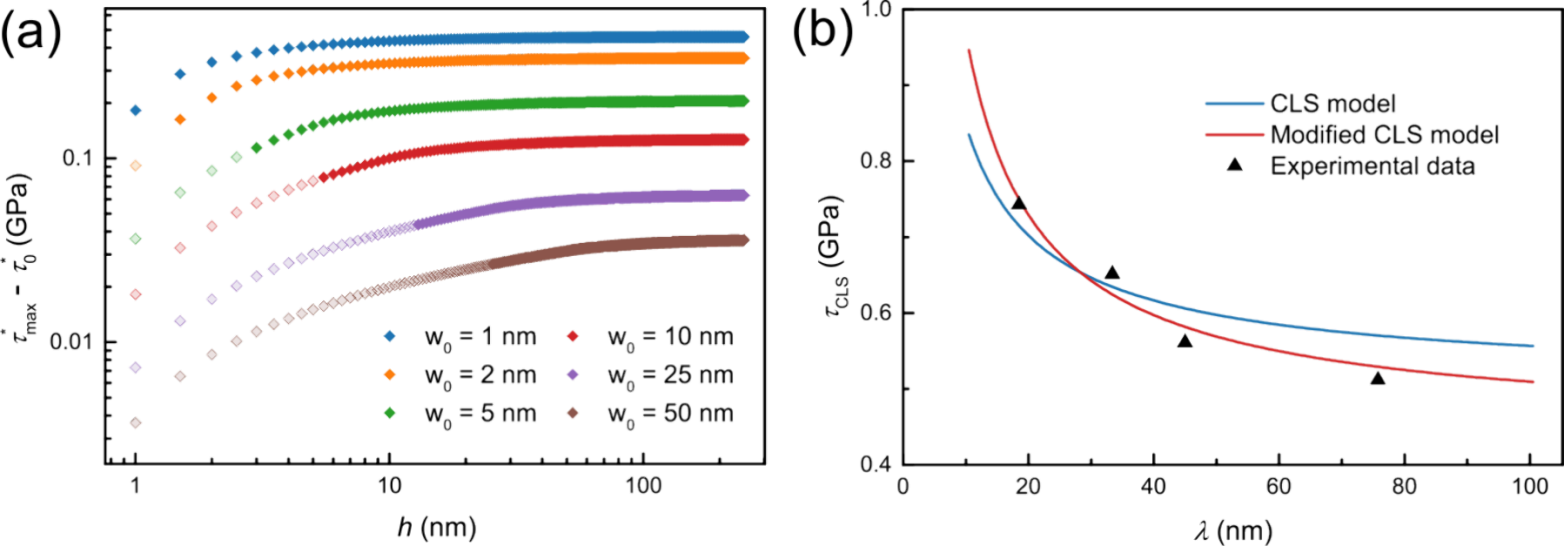
(a) Critical shear stress enhancement τ_max_^*^ – τ_0_^*^ vs *h*, based on the model by Chu and Barnett^15^ for various interface widths. Lighter-colored symbols indicate *w* > *h*, where the peak composition in the layers is below 1. (b) Comparison of the experimental data with predictions from the original and modified CLS models.

Deformation behavior depends on yield differences and the microstructures of Al and Ni layers. Reported data^48,49^ indicate that Al is yielded before Ni, causing dislocation pile-ups at Al/Ni interfaces and Al grain boundaries. For λ ≥ 50 nm, once the pile-up stress reaches τ*, dislocations transmit across the interface or initiate deformation in Ni.^1,20^

TEM reveals submicrometer columnar grains in Al and nanocrystalline grains (<20 nm) in Ni, consistent with vacuum-deposited film behavior governed by *T*/*T*_m_, where *T* is deposition temperature and *T*_m_ is the melting point. At *T*/*T*_m_ < 0.25 (∼0.3), tapered grains form; between 0.3 and 0.45, columnar grains dominate.^50^ Here, *T*/*T*_m_ is 0.17 for Ni layers and 0.31 for Al. The fine Ni grain size limits dislocation pile-ups, making τ* the critical yield stress, i.e., the interface barrier strength.

The interface barrier strength can be estimated from the Hall–Petch slope *k*:^19,21^

2 $$k=a{\left(\frac{\tau^{*}\mu b}{\pi \left(1-\nu \right)}\right)}^{0.5}$$

where μ, *b*, and ν are the shear modulus, the length of the Burgers vector, and Poisson’s ratio of Al, respectively. The constant *a* = 3 follows from the Tabor relation, σ = *H*/3, with *H* as hardness and σ as yield stress. Table 1 lists material parameters.

**Table 1:** Material Parameters for Ni and Al

Material	Shear modulus^51^	Poisson’s ratio^51^	Burgers vector^52^
Ni	76.0 GPa	0.312	0.249 nm
Al	26.1 GPa	0.345	0.286 nm

Using measured Hall–Petch slopes (*k* = 14.7 and *k* = 21.4 GPa $\cdot \sqrt{\mathrm{nm}}$ , Figure 3), τ* is 0.70 and 1.49 GPa, respectively. An alternative estimate using *k* = 0.18 μ√*b*, i.e., considering the critical barrier stress of dislocation pileups as the athermal limit for homogeneous dislocation nucleation,^53^ gives *k* = 22.0 GPa $\cdot \sqrt{\mathrm{nm}}$ with μ from Ni and *b* from Al,^54^ consistent with experiments.

The Hall–Petch model breaks down near λ = 50 nm, where the strength peaks.^19,21^ For λ < 50 nm, dislocations propagate as “hairpin” loops confined within layers, known as confined layer slip (CLS).^19^ The critical resolved shear stress for CLS, τ_CLS_, is

3 $$\tau_{CLS}=\frac{2E\cos\theta }{b\lambda }+\frac{C}{D}$$

with E as the dislocation line energy, θ as the angle between the slip plane and interface, *C* = μ*b*/(1 – ν), and *D* as the spacing between misfit dislocations. Due to the high lattice misfit of Al/Ni and broad interfaces resulting in widely dispersed misfit dislocations,^45^ the *C*/*D* term representing the interaction between CLS and misfit dislocations^19^ is significant. Line energy E is given by

4 $$E=\alpha \mu b^{2}\ln\left(\frac{\lambda }{b\cos\theta }\right)$$

with $\alpha =\frac{1}{4\pi }$ . Experimental shear stress is τ = σ/3.06, with σ = *H*/3 and 3.06 as the Taylor factor.

Equations 3 and 4 are used to fit data for 10 nm< λ < 50 nm, using *D* as a fitting parameter. Since grain size is smaller than layer thickness, λ is taken as the grain size. When CLS dislocations glide along columnar grain boundaries, E reflects the line energy along those paths.

Differences between model (*D* = 41 nm) and experimental data for 10 nm < λ < 50 nm (Figure 4b) likely stem from intermetallic bonding at Al/Ni interfaces, which alters the dislocation energy. To account for this, an additional fitting parameter $E_{i}$ is introduced, modifying the critical resolved shear stress as

5 $$\tau_{CLS}=\frac{2\left(E+E_{i}\right)\cos\theta }{b\lambda }+\frac{C}{D}$$

Figure 4b shows the fit with $E_{i}$ = 0.75 × 10^–9^ J/m and *D* = 47 nm. The modified CLS model describes the experimental data well for 10 nm < λ < 50 nm. The value of $E_{i}$ is comparable to E ≈ 1 × 10 ^–9^ J/m, validating its physical relevance. The seemingly large *D* may reflect interface interdiffusion and the transition from a 2D to a 3D misfit dislocation network, consistent with observations of self-organized interfacial structures in wide interfaces.^55^ Notably, this modified model is also applicable to multilayer structures that apply the original CLS model as they share the same underlying assumptions. Extending its applicability, however, primarily hinges on determining the energy of the trailing dislocations at diverse interfaces.

Understanding the role of the negative enthalpy of mixing is essential. First-principles calculations by Kong and Shen^26^ suggest that strong negative enthalpy may lower interface energy, promoting intermetallic formation. Our experimental results confirm intermetallic bonding at Al/Ni multilayers, which likely increases hardness due to their higher modulus relative to pure Al or Ni. Additionally, new interfaces between intermetallics and base metals introduce extra strengthening mechanisms.

Thus, intermetallic bond formation, driven by the negative enthalpy of mixing, significantly contributes to the high peak hardness observed in such multilayer systems.

Microstructural and compositional analyses of Al/Ni multilayers reveal extensive interdiffusion (25–50 nm interface width) and intermetallic bonding at interfaces and grain boundaries. These multilayers achieve a peak hardness of 9.5 GPa—the highest reported for fcc/fcc systems. Hall–Petch analysis confirms strong interface barrier stresses, although modulus mismatch strengthening remains limited due to interdiffusion. A modified CLS model, incorporating added dislocation line energy, fits the experimental data well, likely reflecting intermetallic bond formation. Additionally, a strong correlation is observed between normalized peak hardness and negative enthalpy of mixing (Figure 5), highlighting intermetallic compound formation as a key factor in strengthening multilayers with negative mixing enthalpies.

**Figure 5:**
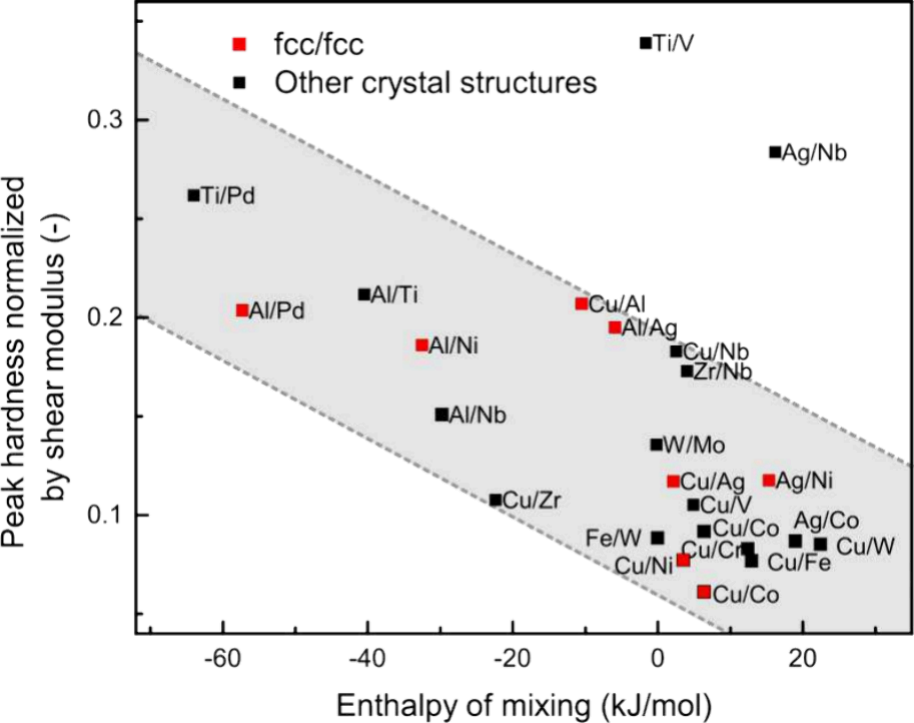
Relation between peak hardness (normalized by the shear modulus) and enthalpy of mixing for various metallic multilayers. Dashed lines and the shaded region serve as visual guides. Data compiled from the literature: Ag/Al,^18^ Cu/V,^37^ Cu/Nb,^37^ Cu/Cr,^37^ Cu/W,^37^ Ag/Nb,^37^ Cu/Fe,^10^ Ag/Ni,^38^ Al/Pd,^23^ Cu/Al,^22^ Cu/Co,^39^ Cu/Ni,^1^ Cu/Ag,^1^ Ag/Co,^9^ W/Mo,^9^ Ti/Pd,^25^ Ti/V,^25^ Fe/W,^40^ Cu/Zr,^41^ Zr/Nb,^11^ and Al/Ag.^18^

## Supplementary Material



## Notes

Further details on materials and methods, detailed XRD and TEM characterization data, and nanoindentation results (PDF)
