# Supplementary material for: Strengthening Mechanism of Al/Ni Multilayers with Negative Enthalpy of Mixing
Source: Nano Lett. 2025 Aug 19;25(34):12914–20. doi: 10.1021/acs.nanolett.5c02939 (PMC12395473; doi:10.1021/acs.nanolett.5c02939)
Supplement: Supplementary file 1 [file nl5c02939_si_002.pdf]

Supporting Information for

Strengthening mechanism of Al/Ni multilayers with negative enthalpy of mixing

Xi Li[1], Nicolas J. Peter[1], Marilaine Moreira de Lima[1], Peter Schaaf [2], and Ruth Schwaiger[*,1]

[1]Institute of Energy Materials and Devices, Structure and Function of Materials (IMD-1), Forschungszentrum Jülich GmbH, 52425 Juelich, Germany ,

[2]Chair Materials for Electrical Engineering and Electronics, Institute of Materials Science and Engineering, Institute of Micro and Nanotechnologies MacroNano, TU Ilmenau, Gustav-Kirchhoff-Str. 5, 98693 Ilmenau, Germany,

[*]Corresponding author, E-mail address: r.schwaiger@fz-juelich.de

1 Materials and Methods

Al/Ni multilayer thin films were prepared by the alternating deposition of Al and Ni layers on Si wafers with a 250 nm thick SiO_2 passivation using DC magnetron sputtering. To minimize oxidation, Ni constituted the topmost layer of each multilayer stack. Sputtering was conducted at room temperature with a power of 200 W, achieving average deposition rates of 0.32 nm/s for Ni and 0.2 - 0.4 nm/s for Al. The chamber was evacuated to a base pressure to 5×10^{-7} mbar prior to sputtering and the Ar pressure was 5×10^{-3} mbar during sputtering. No heat treatment was applied during or after deposition to prevent reactions between Al and Ni. The target materials had purities of 99.98% (Ni) and 99.999% (Al).

2 Microstructure Characterization

X-ray diffractograms of Al/Ni multilayers with varying h are presented in Figure 1a. Aside from small peaks near 54.5°, attributed to the substrate, the diffractograms primarily exhibit diffraction peaks from Al and Ni. The most prominent peak in each profile corresponds to the combined Al(2 0 0) and Ni(1 1 1) reflections around 44°, which are too close in position to be

resolved separately. For samples with h ranging from 10 nm to 250 nm, the relative intensity of the combined Ni(1 1 1)/Al(2 0 0) peak and the Ni(2 2 0) peak is higher compared to other peaks, indicating a mild texture along these orientations. By contrast, the 5 nm sample exhibits significantly higher relative intensities of the Ni(1 1 1)/Al(2 0 0) and Ni(2 2 0) peaks, suggesting a much stronger texture in this sample. Peak broadening is observed as h decreases, indicating a reduction in grain size with decreasing layer thickness. A closer view of the Ni(1 1 1)/Al(2 0 0) and Ni(2 2 0) peaks, shown in Figure 1b, reveals a leftward shift for the 5 nm sample compared to samples with larger h. This shift suggests lattice distortion, likely caused by constraints imposed by the interfaces between the individual layers. Assuming identical orientations of Al and Ni at the interface, the lattice misfit calculated from X-ray diffraction (XRD) peak positions is 13.0%. Therefore, the Al/Ni interface here is incoherent. For $h \leq 10$ nm, lattice constraint effects reflected by peak shifts in XRD slightly reduce the misfit.

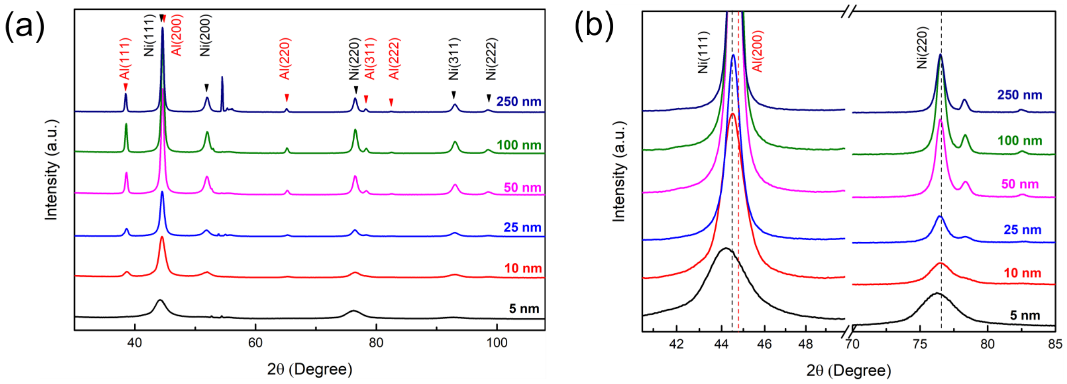

Figure 1: (**a**)XRD patterns of Al/Ni multilayer thin films with layer thickness ranging from 5 nm to 250 nm. (**b**) Enlarged view of the Ni(111)/Al(200) and Ni(220) peaks, showing peak broadening and peak shift in samples with layer thicknesses $h \leq 10$ nm.

To further investigate the microstructures, transmission electron microscopy (TEM) analysis was performed. Figures 2a–c show cross-sectional high-angle annular dark-field scanning TEM (HAADF-STEM) micrographs of the 5 nm, 25 nm and 100 nm Al/Ni multilayers accompanied by their respective selected area electron diffraction (SAED) patterns. These images reveal a modulated layered structure with alternating Ni (bright) and Al (dark) layers, consistent with the Z-contrast in HAADF imaging. The Al/Ni interfaces are sharply defined across all samples, regardless of h.

The SAED patterns of samples with $h \leq 10$ nm exhibit smeared diffraction spots,appearing regularly every 60°, indicative of strong texture. By contrast, samples with $h \geq 10$ nm exhibit ring-like patterns characteristic of polycrystalline microstructures with small grain sizes. For $h > 50$ nm, the SAED patterns increasingly transition to discrete diffraction spots, suggesting larger grains with increasing layer thickness. Integrated radial intensity profiles extracted from

the SAED patterns (Figure 2d) reveal broad peaks corresponding to Al(1 1 1), Ni(1 1 1)/Al(2 0 0), and Ni(2 0 0), consistent with the XRD results. For $h \leq 10$ nm, the profiles indicate strong texture, while for $h = 25$ nm, most of the peaks are clearly visible. For $h = 250$ nm, the profiles lose definition, reflecting dominance by larger grains.

Figure 2e summarizes the structural feature size (λ), including the measured layer thicknesses and grain sizes of Al and Ni layers. The Ni layers closely match their nominal thickness, while Al layers are approximately 15% thinner than intended. Al layers predominantly exhibit columnar grains spanning their entire thickness, with grain width increasing with h up to a maximum of 75 nm for $h = 250$ nm. Conversely, Ni layers are composed of equiaxed grains with diameters below 25 nm, decreasing further for smaller h. Across all samples, microscopy confirmed the absence of pores, indicating high density of the multilayer films.

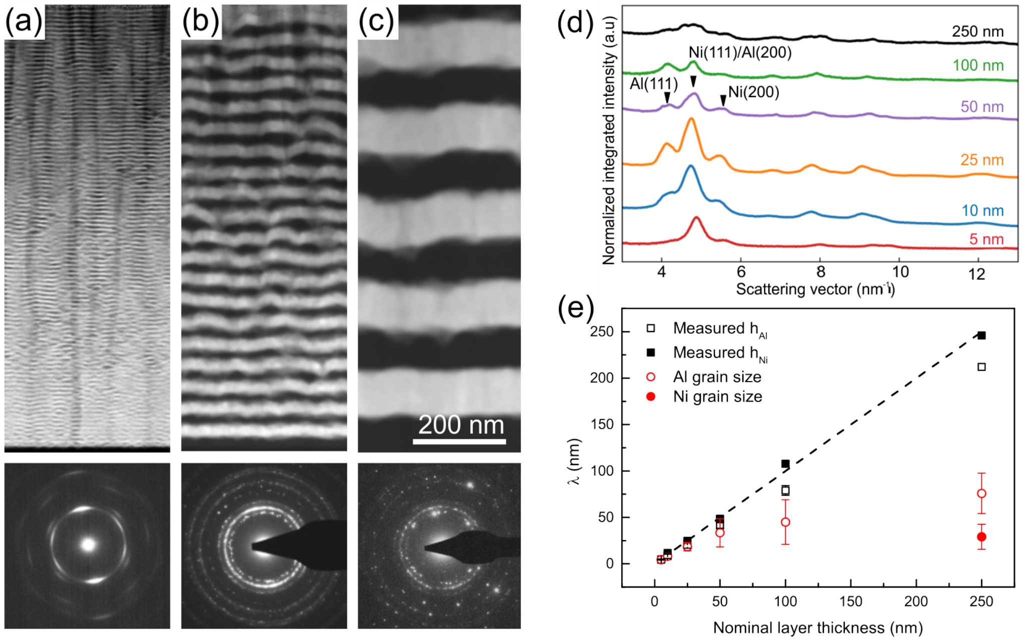

Figure 2: Cross-sectional HAADF-STEM micrographs of (**a**) 5 nm, (**b**) 25 nm and (**c**) 100 nm Al/Ni multilayers with corresponding SAED patterns below showing strong texture in the 5 nm sample and a more random structure in the 25 and 100 nm samples. (**d**) Integrated radial intensity profiles extracted from SAED patterns for $h = 5$–250 nm indicating strong Ni(111) or Al(200) texture for $h \leq 10$ nm consistent with the XRD data. (**e**) Structural feature size, λ, of Al and Ni layers as function of nominal layer thickness.

3 Mechanical properties

The mechanical properties of Al/Ni multilayers were characterized via nanoindentation. Scanning electron microscopy of the residual indents, formed at depths ranging from 100 nm to 200 nm, showed no evidence of material pile-up around indents. This absence of pile-up forma-

tion and, thus, residual stress effects ensures the reliability of the nanoindentation measurements, as these factors are known to cause overestimation of hardness and elastic modulus values [1].

References

[1] Q N Meng, M Wen, C Q Hu, S M Wang, K Zhang, J S Lian, and W T Zheng. Influence of the residual stress on the nanoindentation-evaluated hardness for zirconiumnitride films. *Surface and Coatings Technology*, 206(14):3250–3257, 2012.
